# Therapeutic siRNA for drug-resistant HER2-positive breast cancer

**DOI:** 10.18632/oncotarget.7409

**Published:** 2016-02-15

**Authors:** Shenda Gu, Zhi Hu, Worapol Ngamcherdtrakul, David J. Castro, Jingga Morry, Moataz M. Reda, Joe W. Gray, Wassana Yantasee

**Affiliations:** ^1^ Department of Biomedical Engineering, Oregon Health & Science University, Portland, Oregon, 97239, USA; ^2^ PDX Pharmaceuticals, LLC, Portland, Oregon, 97239, USA

**Keywords:** siRNA, HER2, trastuzumab resistance, breast cancer, nanoparticles

## Abstract

HER2 is overexpressed in about 20% of breast cancers and contributes to poor prognosis. Unfortunately, a large fraction of patients have primary or acquired resistance to the HER2-targeted therapy trastuzumab, thus a multi-drug combination is utilized in the clinic, putting significant burden on patients. We systematically identified an optimal HER2 siRNA from 76 potential sequences and demonstrated its utility in overcoming intrinsic and acquired resistance to trastuzumab and lapatinib in 18 HER2-positive cancer cell lines. We provided evidence that the drug-resistant cancer maintains dependence on HER2 for survival. Importantly, cell lines did not readily develop resistance following extended treatment with HER2 siRNA. Using our recently developed nanoparticle platform, systemic delivery of HER2 siRNA to trastuzumab-resistant tumors resulted in significant growth inhibition. Moreover, the optimal HER2 siRNA could also silence an exon 16 skipped HER2 splice variant reported to be highly oncogenic and linked to trastuzumab resistance.

## INTRODUCTION

Breast cancer is one of the leading causes of death among women in the United States. Among various subtypes of breast cancer, overexpression and amplification of the human epidermal growth factor receptor type 2 (HER2; *ErbB2/neu*) accounts for about 20% of all cases and is a predictor of aggressive phenotype and poor prognosis [1]. HER2, a member of the HER family, is a transmembrane receptor tyrosine kinase and has been well characterized as an oncogenic driver of human breast and ovarian cancer [2]. Activation of HER2 downstream signaling requires either self-dimerization or dimerization with other HER family members [3]. Downstream signaling is mainly mediated through activation of AKT and ERK pathways, leading to cellular proliferation and survival [4]. Overexpression of HER2 increases downstream activity by increasing the probability of homo- and heterodimer formation [5] and/or by increasing the production of a highly oncogenic HER2 splice variant that lacks exon 16 hereafter referred to as delta16 HER2 [6].

FDA-approved targeted therapies for HER2-positive breast tumors include trastuzumab (Herceptin^®^, Genentech), pertuzumab (Perjeta^®^, Genentech), T-DM1 (Kadcyla^®^, Genentech) and lapatinib (Tykerb^®^, Norvatis). Trastuzumab and pertuzumab are humanized monoclonal antibodies that bind to the extracellular domain of the HER2 receptor and block receptor dimerization, thus preventing receptor activation [7]. T-DM1 is trastuzumab conjugated to the cytotoxic agent emtansine. Lapatinib is a small molecule dual kinase inhibitor that actively blocks HER2 signaling by binding to the HER2 and/or EGFR kinase domain [8]. Despite the use of the aforementioned HER2-targeted therapies, patients with advanced HER2-positive breast cancer still develop resistance to the best combination regimens (e.g., trastuzumab, pertuzumab, and docetaxel) and progression-free survival is still only 18.5 months [9]. Clearly, more durable treatments are needed.

Recent studies have shown the potential use of HER2 siRNA as therapeutics for treating HER2-positive breast cancer [10, 11]. One group has demonstrated its potential to overcome resistance to trastuzumab [12]. However, these works have been limited to in vitro studies due to the lack of readily available in vivo delivery platforms. In addition, the siRNA sequences used in prior works have not been systematically screened. Some studies have infected cancer cells ex vivo with HER2 siRNA [13] or shRNA [14] prior to tumor inoculation in order to demonstrate the in vivo activity of HER2 suppression. However, to make RNAi clinically relevant, effective in vivo delivering of siRNA to tumors is necessary. Although viral-based siRNA and shRNA strategies are effective, concerns regarding immunogenic response and insertional mutagenesis remain major issues [15]. Recent advances in nanobiotechnology have made non-viral based siRNA delivery viable. Inoue et al. showed that a polymalic acid-based nanobiopolymer conjugated with HER2 antisense and trastuzumab can inhibit tumor growth in BT474 tumor xenografts [16]. Two additional studies have utilized siRNA against PLK1 delivered systemically with peptide fusion protein [17] or PLA-PEG [18] to successfully treat BT474 tumors in mice. However, these studies utilized BT474 derived tumors, which are sensitive to trastuzumab.

This article addresses several aforementioned shortcomings in the field. We have identified the most optimal HER2 siRNA duplex from a pool of 76 potential sequences. We then demonstrate the in vitro efficacy of the optimal HER2 siRNA to overcome both intrinsic and acquired drug resistance in HER2-positive cancer cell lines, followed by the in vivo efficacy by utilizing our recently optimized nanoparticle platform [19] to systemically deliver siRNA to solid tumors. We elucidate that the drug-resistant cancer still relies on HER2 pathways, substantiating the utility of a HER2 siRNA treatment strategy to overcome drug resistance. Importantly, we also address whether HER2-positive cancer can develop resistance to HER2 siRNA after long-term treatment as it does with HER2-targeted therapies. Lastly, we show that our optimal HER2 siRNA can silence both wild-type HER2 and the more oncogenic delta16 HER2 splice variant to the same extent.

## RESULTS

### Screening and validation of HER2 siRNA

**Figure 1:**
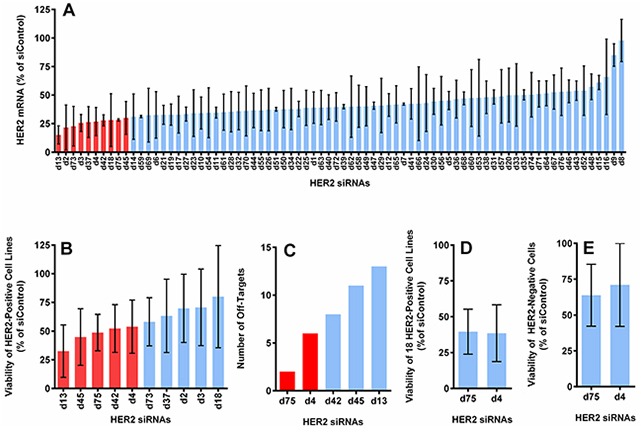
Selection of optimal HER2 siRNA sequences based on the level of HER2 mRNA knockdown, cell viability reduction, number of off-targets and specificity **A.** The average expression of HER2 mRNA in 3 HER2-positive cell lines (BT474, SKBR3 and HCC1954), measured by QuantiGene assay at 5 days post-transfection (normalized to actin and reported as the percentage of the average of 4 control siRNAs). **B.** The cell viability reduction induced by the top 10 candidates from (A), measured with CellTiter-Glo assay at 5 days post-transfection. Graph shows the average value from 5 HER2-positive cell lines (BT474, SKBR3, HCC1954, HCC1569, and JIMT1). **C.** The number of off-targets found through BLAST for the top 5 sequences from (B). **D.** Cell viability reduction induced by the best 2 sequences from (C) in 18 HER2-positive cell lines (see Supplementary Figure S1). **E.** Cell viability reduction induced by the best 2 sequences in 2 HER2-negative cell lines (T47D and MCF10A). All with 10 nM of siRNA delivered with DharmaFECT-1.

76 potential HER2 siRNA candidates plus 2 scrambled siRNAs, an siRNA against luciferase and an siRNA against GFP, were tested for their HER2 mRNA knockdown efficiency in the HER2-positive cell lines BT474, SKBR3 and HCC1954. The 76 siRNAs against HER2 were designed to target sequences distributed across the whole coding region of the human ErbB2 gene (NM_001005862). All siRNAs had 3′ UU overhangs and no additional modifications. The sequences of all HER2 siRNAs and control siRNAs are listed in Supplementary Tables S1 and S2. The HER2 mRNA levels in HER2-positive breast cancer cell lines (BT474, SKBR3, and HCC1954) were measured following 5 days of transfection with 10 nM of each individual HER2 siRNA sequence (Figure 1A).

We retested the ability of the ten sequences with the highest knockdown efficiency to inhibit growth of BT474, SKBR3, HCC1954, HCC1569, and JIMT1 (Figure 1B). The five most effective sequences from this evaluation were further ranked according to the number of off-target matches by utilizing BLAST (BLASTN 2.2.31+, RefSeq, NCBI Reference Sequence Database) with a minimum threshold of 70% sequence homology (Figure 1C). The sequences d75 and d4 had the fewest off-target matches and were further validated in a larger panel of 18 HER2-positive cell lines (Figure 1D and Supplementary Figure S1A) and 2 HER2-negative cell lines (Figure 1E). Both sequences yielded comparable cell killing in the 18 cell lines and showed similar treatment specificity (i.e., were less likely to kill HER2-negative cells). However, d75 had fewer off-target matches than d4 (2 vs. 6) and d75 generated more consistent mRNA knockdown and cell viability reduction among the HER2-positive cell lines tested. Low off-target was also apparent when tested in T47D (HER2-negative) cell line, in which GI50 was not achieved even when the concentration of d75 was increased to 80 nM (Supplementary Figure S1B). We selected sequence d75 for further development based on these results. Henceforth, d75 will be referred to as siHER2^d75^ and the AllStar non-targeting control siRNA will be referred to as siControl (Supplementary Table S2).

**Figure 2:**
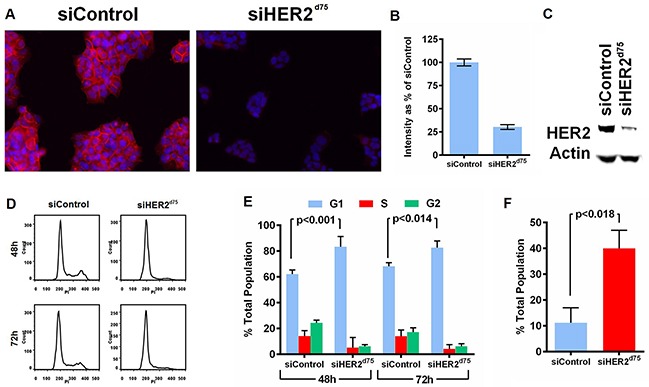
Effect of siHER2^d75^ on HER2 protein expression levels, cell cycle, and apoptosis in the BT474 cell line **A.** HER2 protein reduction by immunofluorescent imaging (200× magification). **B.** Corresponding quantified mean signal intensity of HER2. **C.** HER2 protein reduction analyzed by western blot. Cell were transfected with 10 nM siHER2^d75^ or siControl for 72 h prior to analysis. **D.** Flow cytometry analysis of the treated cells stained with propidium iodide (PI) after 48 h or 72 h. **E.** The corresponding percent distribution of cell cycle analyzed using FlowJo. **F.** The percent apoptotic cells measured by YO-PRO-1 staining of treated cells after 72 h.

The ability of siHER2^d75^ to decrease HER2 protein levels and induce apoptosis and cell cycle arrest was assessed subsequently (Figure 2). Visual and quantitative analyses of the intensity of anti-HER2 immunofluorescence staining before and after treatment with siHER2^d75^ showed that HER2 protein levels on the cellular membrane and in the cytoplasm decreased significantly in BT474 cells after 10 nM siHER2^d75^ treatment for 72 h (Figure 2A–2B). We confirmed the reduction of HER2 protein levels by western blot analysis (Figure 2C). Similar results were also found in SKBR3 and HCC1954 cells (Supplementary Figure S2). The siHER2^d75^ treatment significantly increased the fraction of BT474 cells in the G1 phase of the cell cycle 48 and 72 hours post-treatment compared to siControl (p<0.001 and p<0.014 for 48 and 72 hours, respectively; Figure 2D–2E) and increased apoptotic response by approximately 4-fold over the siControl at 72 h post-transfection (p<0.018; Figure 2F).

### Overcoming intrinsic resistance to trastuzumab and lapatinib with siHER2^d75^

**Figure 3:**
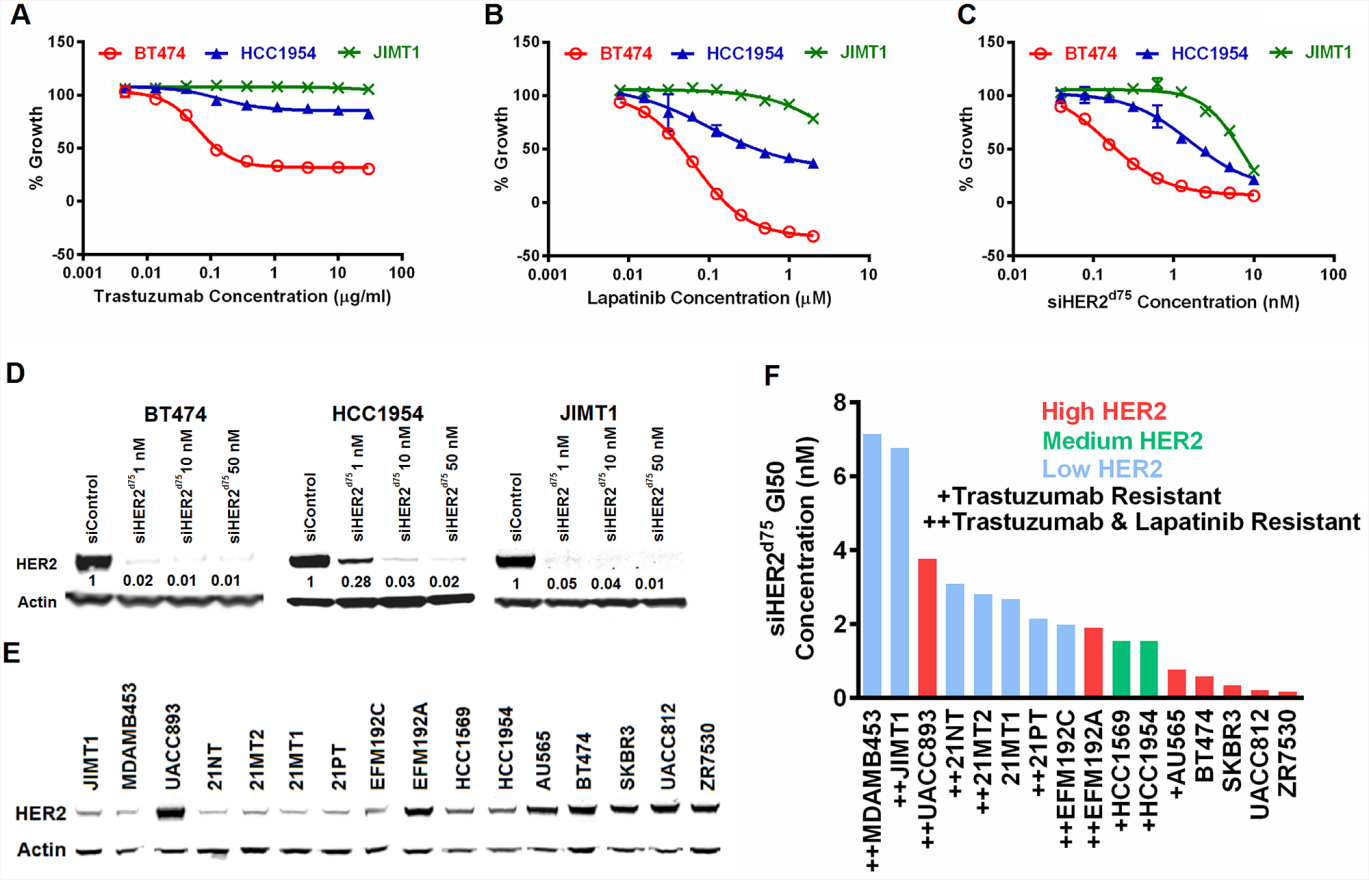
Evaluation of growth inhibition with HER2-targeted therapies in representative breast cancer cells The % Growth of BT474, HCC1954 and JIMT1 breast cancer cell lines treated with **A.** trastuzumab, **B.** lapatinib, or **C.** siHER2^d75^ at the indicated dose range. **D.** Reduction of HER2 protein by various doses of siHER2^d75^ vs. 10 nM of siControl after 72 h. Indicated values in (D) represent the fraction of HER2 remaining relative to siControl. **E.** Western blot comparing the expression levels of HER2 in 16 HER2-amplified cell lines. Samples were run on 3 separate gels and scanned blots were later combined. **F.** Concentration of siHER2^d75^ required to inhibit growth by 50% (GI50) in the 16 cell lines at 5 days post-treatment. “Trastuzumab-resistant” defined as inability to achieve at least 20% growth inhibition at 30 μg/ml dose; “Lapatinib-resistant” defined as inability to achieve at least 20% growth inhibition at 2 μM.

BT474, HCC1954 and JIMT1 breast cancer cell lines were used to benchmark responses to HER2-targeted therapies because they exhibit different sensitivities to trastuzumab and lapatinib in vitro. They also represent HER2 amplification in both the luminal and basal subtypes. The concentrations of trastuzumab and lapatinib needed to inhibit growth by 50% (GI50) in Figure 3A–3B are summarized in Table 1. BT474 was sensitive to both trastuzumab and lapatinib with GI50 values of 0.12 μg/ml and 0.05 μM, respectively. However, JIMT1 was highly resistant to both HER2-targeted therapies, and HCC1954 showed a marginal response to lapatinib (GI50 of 0.39 μM) and was largely resistant to trastuzumab. Although lapatinib showed some ability to inhibit growth in all three cell lines, the dose required to inhibit the resistant cell lines (HCC1954 and JIMT1) exceeded 2 μM, which starts to be toxic to some HER2-negative cells [20]. Not surprisingly, differential sensitivity to siHER2^d75^ was also observed across the three cell lines (Figure 3C, with representative HER2 reduction in Figure 3D) as evident in the calculated GI50 values, which ranged from 0.19 to 6.78 nM (Table 1). However, an siHER2^d75^ dose of only 10 nM could inhibit the growth of BT474, HCC1954, and JIMT1 by 95%, 80%, and 70%, respectively. This suggests that the intrinsically resistant HCC1954 and JIMT1 cell lines still require HER2 for growth, and silencing of HER2 using siRNA in the low nanomolar range is a viable therapeutic strategy.

**Table 1:** GI50 concentrations of trastuzumab, lapatinib and siHER2^d75^

Cell Lines	Trastuzumab (μg/ml)	Lapatinib (μM)	siHER2^d75^ (nM)
JIMT1	>30	>2	6.78 (5.97, 7.60)
HCC1954	>30	0.39 (0.27, 0.58)	2.15 (1.81, 2.60)
BT474	0.12 (0.11, 0.14)	0.05 (0.045, 0.049)	0.19 (0.18, 0.21)
BT474-TR	>30	0.13 (0.12, 0.14)	0.27 (0.20, 0.34)
BT474-LR	>30	1.01 (0.89, 1.15)	0.75 (0.66, 0.87)

Lower and upper limits of 95% CI are given in parentheses.

Previous studies have reported a positive correlation between the efficacy of trastuzumab and lapatinib and the elevated protein expression of HER2 [20, 21]. We found that response to siHER2^d75^ in BT474, HCC1954 and JIMT1 was also proportional to HER2 protein expression levels (Figure 3E). We investigated the correlation between HER2 protein levels and siRNA induced growth inhibition by measuring GI50 values for siHER2^d75^ in 16 HER2-positive cell lines with varied HER2 levels (Figure 3E), many of which were resistant to trastuzumab or both trastuzumab and lapatinib. Indeed, the HER2 protein levels of the 16 cells were largely predictive of their response to siHER2^d75^ as shown in Figure 3F. One exception was the UACC893 cell line, which has the highest HER2 expression but only moderately responded to siHER2^d75^ treatment. One possible explanation is that this cell line harbors a PIK3CA^H1074R^ mutation, which can result in AKT activation independent of HER2 [22]. Nonetheless, the GI50 values for all these cell lines ranged between 0.15 and 7 nM, thus still supporting the potency of siHER2^d75^ in achieving growth inhibition. In general, HER2 protein level was more predictive of the response to siHER2^d75^ than HER2 mRNA level generated with RNAseq [23] (Supplementary Figure S3).

### Overcoming acquired resistance to trastuzumab and lapatinib with siHER2^d75^

**Figure 4:**
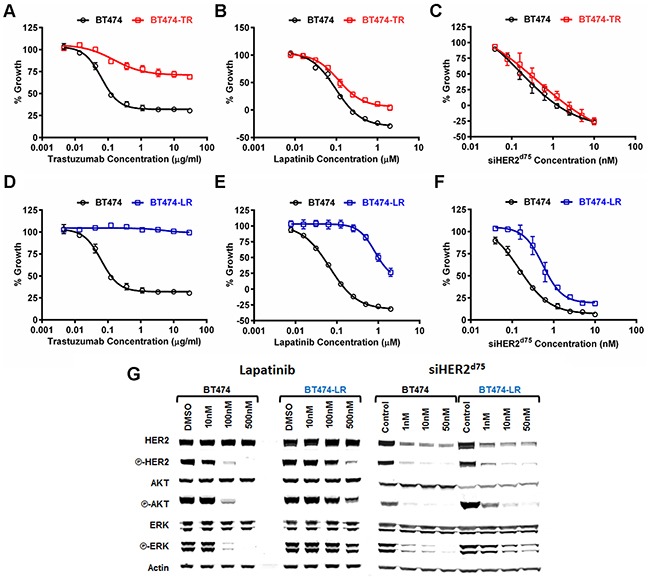
Dose response characterization of parental BT474 and BT474 with acquired resistance to trastuzumab (BT474-TR) or lapatinib (BT474-LR) to HER2-targeted agents **A–C.** Growth inhibitory responses of BT474-TR to trastuzumab, lapatinib, and siHER2^d75^, respectively. **D–F.** Those on BT474-LR. Cell growth was measured at 3 days post-treatment with lapatinib, or 5 days post-treatment with trastuzumab or siHER2^d75^. **G.** Effects of lapatinib and siHER2^d75^ treatment on HER2 signaling pathway of BT474 and BT474-LR cells. Cells were treated with specified doses of lapatinib or siHER2^d75^ for 3 days and the key signaling molecules were analyzed by western blot.

We used the BT474 cell line to study acquired resistance to trastuzumab and lapatinib. Briefly, BT474 cells were selected in our lab in the presence of increasing concentrations of lapatinib (0.05-1 μM) or trastuzumab (10-30 μg/ml) over a period of 6 months, similar to the method published by Wang et al. [12]. These two cell line derivatives were referred to as BT474-TR for Trastuzumab-Resistant and BT474-LR for Lapatinib-Resistant. We measured the responses of BT474-TR and BT474-LR to trastuzumab, lapatinib or siHER2^d75^. BT474-TR was resistant to trastuzumab (Figure 4A) but remained sensitive to lapatinib (Figure 4B), while BT474-LR was resistant to both trastuzumab (Figure 4D) and lapatinib (Figure 4E). Importantly, we did not observe significant resistance to siHER2^d75^ in these two derivatives (Figure 4C & 4F) although the siHER2^d75^ GI50 did increase from 0.19 nM to 0.75 nM in the BT474-LR cell line (Table 1).

### Impact of siHER2^d75^ and lapatinib on phosphorylation status of HER2, AKT an ERK

Lapatinib inhibits HER2 signaling by binding to the HER2 kinase domain and in so doing, decreases phosphorylation of HER2, AKT and ERK [20]. We assessed phosphorylation status of HER2, AKT and ERK in the lapatinib-resistant BT474-LR cell line in order to gain mechanistic insight into the cellular responses to increasing doses of lapatinib and siHER2^d75^ treatment (Figure 4G). We focused on BT474-LR since it was resistant to both trastuzumab and lapatinib. Untreated BT474-LR displayed a level of phosphorylated HER2 similar to that in the parental BT474. This suggests that BT474-LR cells are still dependent on HER2 signaling. Lapatinib given at 100 nM could block the phosphorylation of HER2, AKT and ERK in the parental BT474 at 72 h post-treatment, whereas a dose of 500 nM was barely able to do so in BT474-LR. On the other hand, 10 nM siHER2^d75^ was effective in BT474-LR displaying reduced phosphorylation of HER2, AKT and ERK to levels that were similar to those achieved in the parental BT474. This correlates well with our finding (Figure 4F) that the sensitivity of BT474-LR to siHER2^d75^ was still on par with that of the parental BT474.

### Effect of extended HER2 silencing on BT474 response to trastuzumab, lapatinib, or siHER2^d75^

We next asked whether HER2-positive cells can develop resistance to siHER2^d75^ after extended treatment. We assessed that by treating BT474, HCC1954 and JIMT1 cells with 1 nM siHER2^d75^ or siControl weekly for 15 weeks. The resulting cell line derivatives were designated as BT474-H15, HCC1954-H15 and JIMT1-H15 (siHER2 for 15 weeks), and BT474-C15, HCC1954-C15 and JIMT1-C15 (siControl for 15 weeks). One nanomolar of siHER2^d75^ was sufficient to reduce the expression of HER2 by approximately 98% in BT474, 72% in HCC1954, and 95% in JIMT1 cells (Figure 3D). When leaving 1 nM siHER2^d75^ in the medium following transfection, the silencing effect as gauged by HER2 mRNA level reduction lasted for at least 7 days (Supplementary Figure S4A). Thus, the siHER2^d75^ transfection was repeated at 7-day intervals when medium change was also needed. Immunofluorescence imaging (Supplementary Figure S4B) confirmed that HER2 reduction occurred uniformly across the BT474 cell population. Because 1 nM siHER2^d75^ did not eradicate the entire cell population, cells that survived under low HER2 conditions were selected over the 15 weeks. After 15 weeks, the remaining cells designated BT474-H15, HCC1954-H15 and JIMT1-H15 were expanded upon the withdrawal of siHER2^d75^ for further characterization. Without siHER2^d75^, the rebound of HER2 expression to the level of the naïve cells (never been treated with siHER2^d75^) was observed within a week (Supplementary Figure S4C).

**Figure 5:**
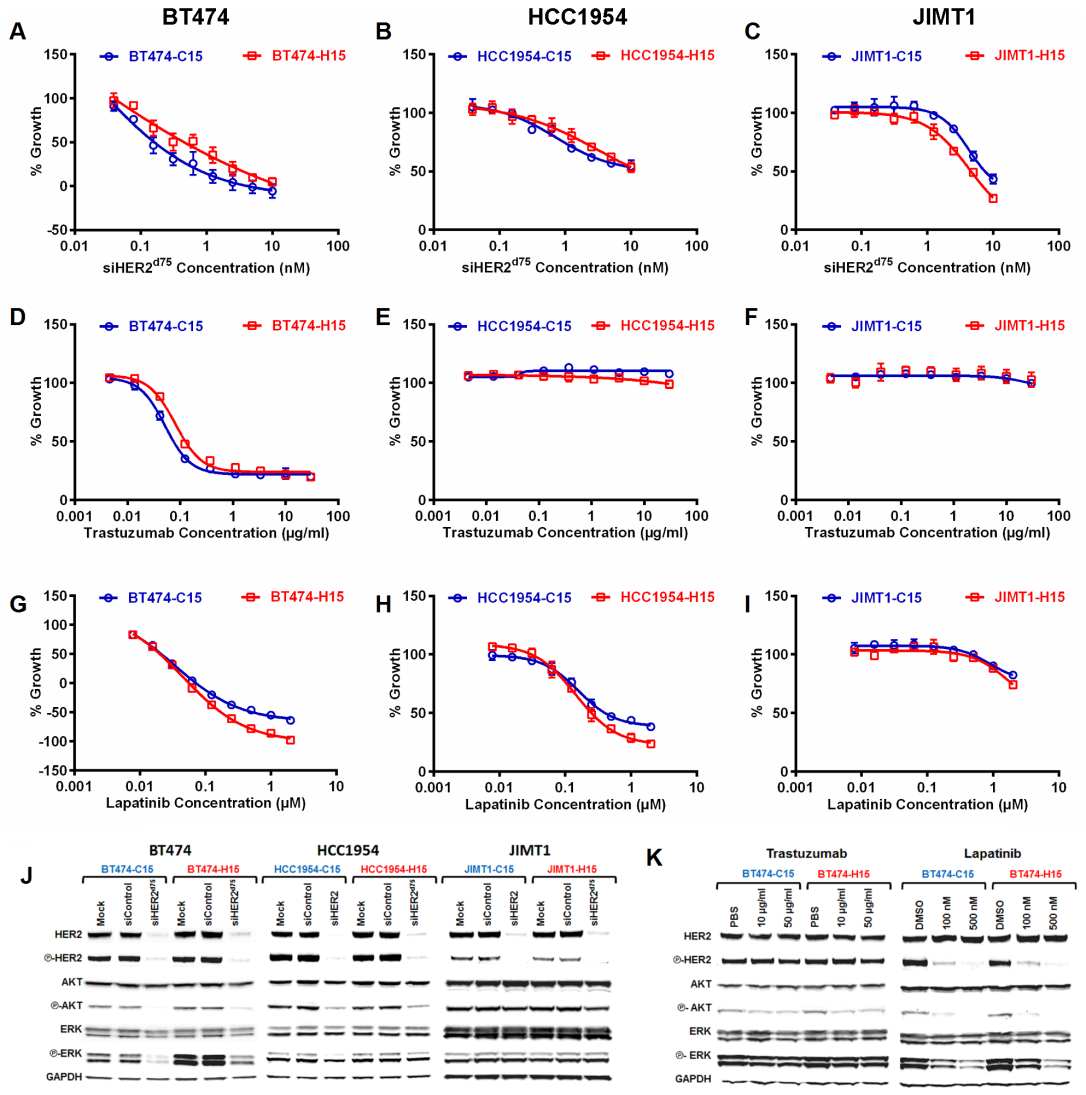
Growth inhibitory effect (5 days) of **A–C.** siHER2^d75^, **D–F.** trastuzumab, and **G–I.** lapatinib in BT474, HCC1954, and JIMT1 cells that had been pre-treated weekly with 1 nM of siHER2^d75^ (H15) or siControl (C15) for 15 weeks. **J.** HER2 signaling molecules in specified cell lines at 3 days after 10 nM siHER2^d75^ treatment. **K.** HER2 signaling molecules in BT474-C15 and BT474-H15 at 3 days after 10–50 μg/ml trastuzumab or 100-500 nM lapatinib treatment.

The responses of the repeatedly transfected cells to varied doses of siHER2^d75^ were not significantly different between the C15 and H15 populations generated from BT474, HCC1954, and JIMT1 (Figure 5A–5C, respectively). Also, the cells had not developed resistance to either trastuzumab (Figure 5D–5F) or lapatinib (Figure 5G–5I) following prolonged siHER2^d75^ treatment, illustrating that cells receiving extended siHER2 treatment still depend on HER2. In contrast, a slight increase in sensitivity to lapatinib especially at the higher dose range was observed with the H15 populations (vs. C15 counterparts) generated from BT474 and HCC1954 (Figure 5G & 5H).

Figure 5J shows the phosphorylation state of signaling molecules in the HER2 pathway of the C15 and H15 populations. The protein profiles of H15 lines before treatment were comparable to those of C15. The apparent slight upregulation of phosphorylation of ERK in BT474-H15 was not significant once normalized to the GAPDH band intensity. Both C15 and H15 cells responded similarly to treatment with siHER2^d75^ in terms of HER2 silencing and inhibition of phosphorylation of HER2, AKT and ERK. Figure 5K shows that lapatinib and trastuzumab were able to inhibit phosphorylation of AKT and ERK in BT474-H15. The effect of trastuzumab was less pronounced than that of lapatinib, which has previously been shown to be more effective at blocking HER2 signaling [12]. Collectively, these results suggest that HER2-positive cancer cells still rely on the HER2 signaling pathway after extended siHER2^d75^ treatment and are much less prone to develop resistance to siHER2^d75^ than to trastuzumab or lapatinib.

### Silencing delta16 HER2 with siHER2^d75^

**Figure 6:**
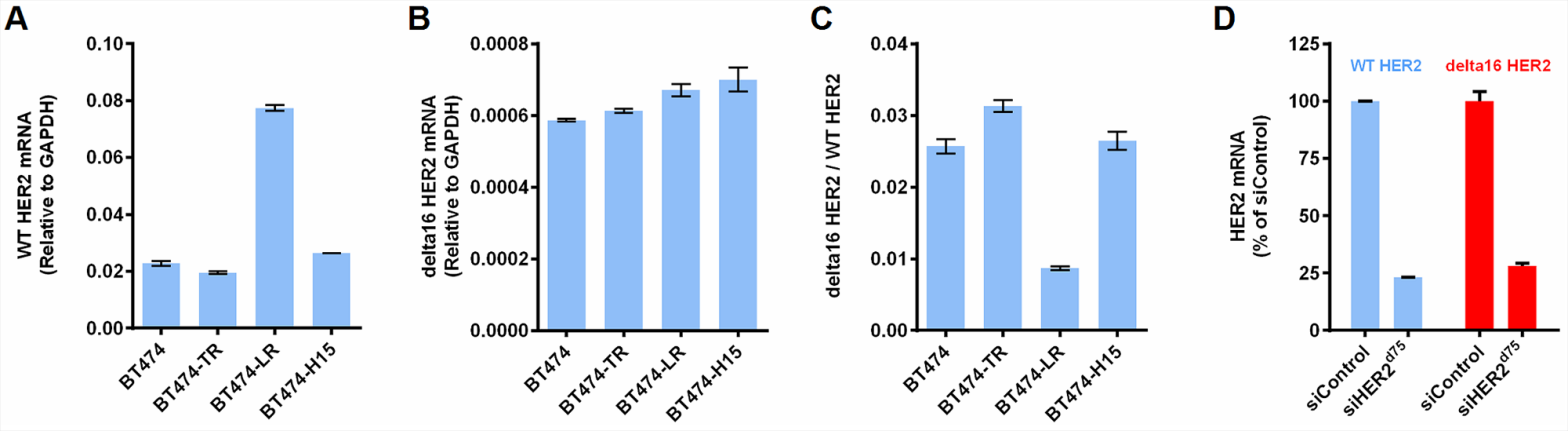
The siHER2^d75^ mediated gene knockdown of the delta16 HER2 splice variant **A.** WT HER2 mRNA levels of parental BT474, BT474-TR, BT474-LR, and BT474-H15. **B.** Delta16 HER2 mRNA of corresponding cells. **C.** Ratios of delta16 HER2 over WT HER2. **D.** % Knockdown of WT HER2 mRNA and delta16 HER2 mRNA in BT474 cells at 48 h post transfection with 10 nM siHER2^d75^ (vs. siControl)

The delta16 HER2 is a more oncogenic variant of HER2 and has been implicated in tumor aggressiveness. Studies have shown that only 5 copies of delta16 HER2 are needed to drive oncogenic transformation, whereas 30-50 copies of WT HER2 were needed to initiate tumorigenesis [24]. Delta16 HER2 has also been implicated in cancer resistance to trastuzumab [25] and tamoxifen [26]. Therefore, the elimination of delta16 HER2 is of great therapeutic value. We hypothesize that since siHER2^d75^ targets a region upstream of exon 16, it should be able to silence both delta16 HER2 and WT HER2 with similar efficacy. Figure 6A–6C shows the mRNA levels of wild-type HER2, delta16 HER2, and their ratios in parental BT474, BT474-TR, BT474-LR, and BT474-H15. A slight increase in the ratio of delta16 HER2 over WT HER2 was observed in BT474-TR, compared to that in parental BT474 (p < 0.01). In BT474-LR, the WT HER2 was upregulated, while delta16 HER2 was not. siHER2^d75^ silenced both HER2 isoforms as shown by similar percent reduction in mRNA levels in BT474 cells at 48 h post-transfection (vs. siControl) (Figure 6D), confirming its broad specificity. This agrees with data from BT474-H15 (receiving a long term treatment of siHER2^d75^) that there is no significant change in delta16 HER2/WT HER2 ratio compared to the parental BT474 cells (Figure 6C).

### Delivery of siHER2^d75^ to HER2-positive breast cancer with targeted nanoconstructs

**Figure 7:**
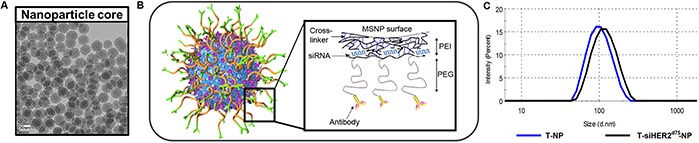
Trastuzumab-conjugated mesoporous silica nanoconstruct **A.** TEM image of the mesoporous silica nanoparticle (MSNP) core (scale bar = 50 nm). **B.** Schematic of surface modification of MSNP (layer-by-layer) with cross-linked polyethylenimine (PEI), polyethyleneglycol (PEG), trastuzumab, and siRNA. **C.** Hydrodynamic size distribution of T-NP (blue line) and with siRNA loading (black line).

To show clinical and translational potential, we systemically delivered siHER2^d75^ to HER2-positive breast tumors with trastuzumab-conjugated mesoporous silica based nanoconstructs (designated “T-NP”) recently reported by us [19]. The silica core has a uniform diameter of 50 nm and is further modified with the addition of layers of polyethylenimine (PEI), polyethyleneglycol (PEG), trastuzumab, and siRNA (Figure 7). The hydrodynamic size of the particles is 104 ± 1.7 nm and the zeta potential is 8.10 ± 0.3 mV in 10 nM NaCl after loading of siHER2^d75^. When delivered with our nanoparticles, siHER2^d75^triggered less or comparable immune response when compared to the FDA-approved nanoparticle based drugs Abraxane and Feraheme [19]. The material given IV could treat intrinsically resistant HCC1954 tumors (significantly after a couple doses) and knock down 60% of HER2 protein in the tumors after one dose [19].

**Figure 8:**
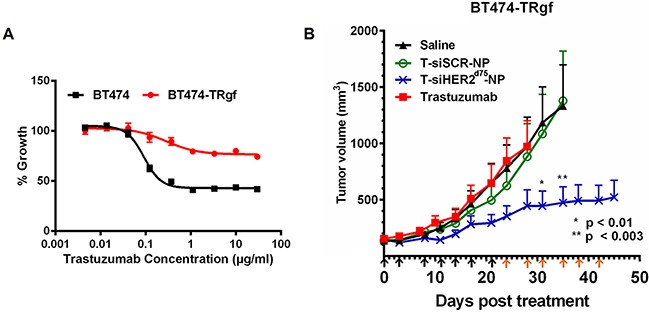
Efficacy of T-siHER2^d75^-NP in BT474-TRgf **A.** In vitro trastuzumab dose-response curve (as % growth 5 days post-treatment) of BT474 and BT474-TRgf. **B.** Mice bearing BT474-TRgf xenografts (n=5-7/group) were injected via tail vein with saline, trastuzumab (2.5 mg/kg given IV, twice weekly) or trastuzumab-conjugated nanoparticles (T-NP) loaded with siHER2^d75^ or siSCR. Arrows below the x-axis indicate nanoparticles injections; black indicate 1.25 mg siRNA/kg; orange, 2.5 mg siRNA/kg. Tumor volumes are presented as means ± SEM. Specified p-values are against the saline control.

Here we show that the T-siHER2^d75^-NP could also treat a BT474 variant with acquired resistance to trastuzumab. The BT474 variant was developed in Kerbel's lab [27] by serial passaging BT474 in mice for 3 years. The resulting tumors grow aggressively in mice without estrogen supplement (unlike the parental BT474 tumors). After 2-6 months of trastuzumab treatment (20 mg/kg twice weekly, IP), the tumor that developed resistance was adapted into tissue culture. We subsequently passaged the cells in mice once more to further select for those that grew aggressively. These new tumors were adapted into culture and named BT474-TRgf. In addition to being selected with trastuzumab in vivo, the BT474-TRgf maintained its resistance to trastuzumab in vitro (Figure 8A). Figure 8B shows that the BT474-TRgf cell grown in mice still responds well to our T-siHER2^d75^-NP treatment, compared to the untreated or T-siSCR-NP. The tumors also showed a better response to our T-siHER2^d75^-NP than free trastuzumab, given IV (twice weekly) at the equivalent dose to that of the nanoconstructs. The fact that tumors were not responsive to T-siSCR-NP also indicates that growth inhibition is due to siHER2, rather than trastuzumab on the nanoparticles.

### Cytotoxicity of T-siHER2^d75^-NP in normal/non-tumorigenic HER2-negative cells

**Figure 9:**
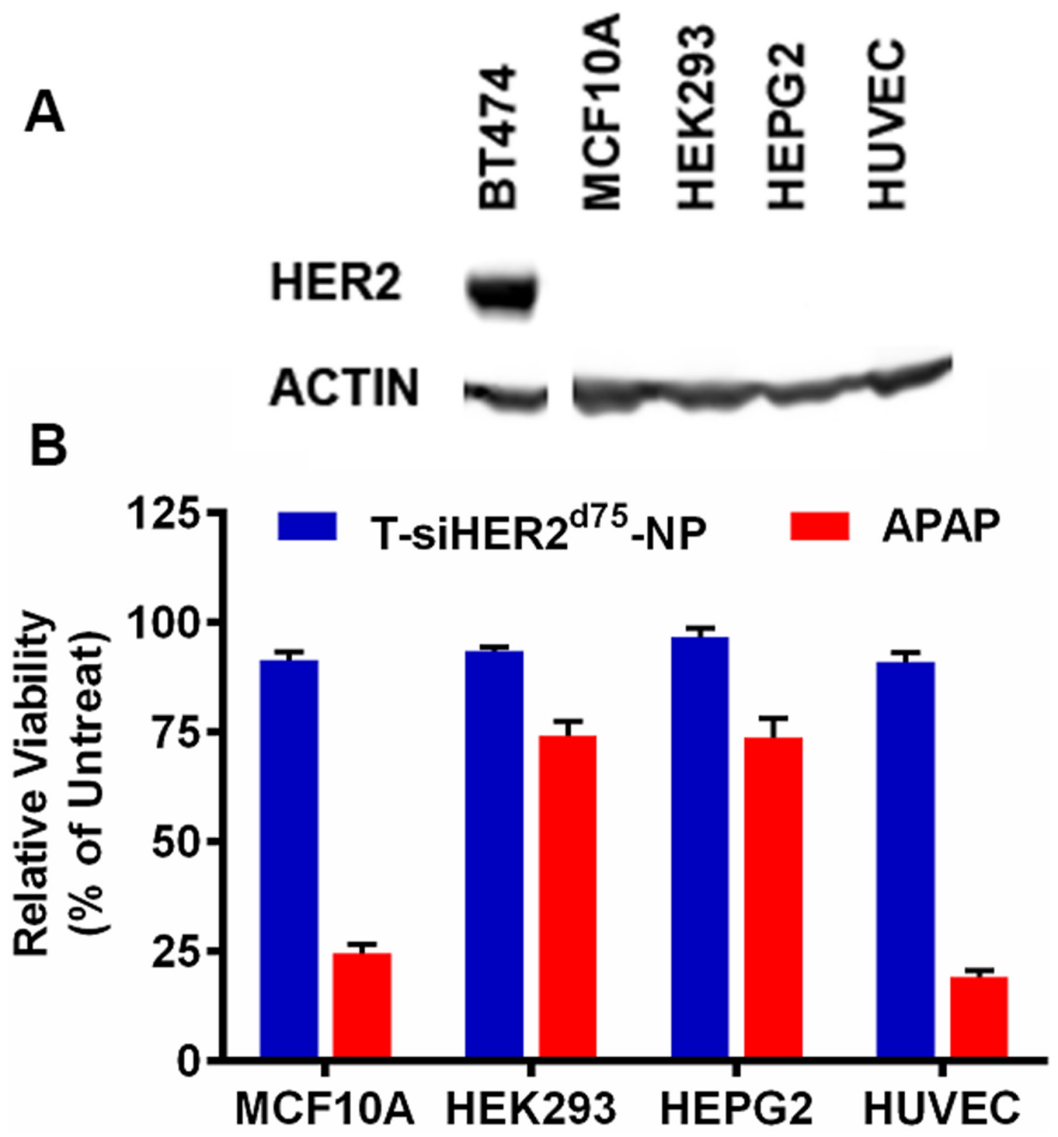
Cytotoxicity of T-siHER2^d75^-NP in a panel of non-tumorigenic cell lines, MCF10A, HEK293, HEPG2, and HUVEC **A.** Western blots indicate no detectable HER2 expression in these cell lines when compared to BT474. **B.** Low cytotoxicity (<10% cell death) of T-siHER2^d75^-NP at efficacious dose (60 nM as siHER2^d75^) compared to the drug benchmark, APAP (25 mM). Cell viability was measured at 24 h after the treatment and reported as the percentage of the untreated control.

We evaluated the cytotoxicity of T-siHER2^d75^-NP in normal/non-tumorigenic (HER2-negative) cells including HEK293 (human embryonic kidney cells), HEPG2 (human liver hepatocellular cells), MCF10A (human mammary epithelial cells), and HUVEC (human umbilical vein endothelial cells) as shown in Figure 9. Kidney and liver cell lines have been recommended by the Nanotechnology Characterization Lab (NCL) of NCI for cytotoxicity studies of nanoparticles since kidney and liver are clearance organs for nanoparticles [28, 29]. MCF10A is considered a normal counterpart of breast cancer, and HUVEC is a relevant cell model for intravenous administration of nanoparticles. Figure 9A shows that these cells have no detectable HER2 expression when compared to BT474. At the efficacious dose for killing cancer cells [19], Figure 9B shows that our T-siHER2^d75^-NP did not elicit significant cytotoxicity in any cell line tested (<10% cell death). T-siHER2^d75^-NP appeared safer than the drug benchmark, APAP, recommended by NCL for nanoparticles' cytotoxicity study.

## DISCUSSION

One major challenge today in treating HER2-positive cancer is the intrinsic and acquired resistance to HER2-targeted therapies. Studies to understand the intrinsic and acquired resistance to HER2-targeted therapies have implicated post-translational variants, splicing variants, and mutations of HER2 as potential mechanisms. One variant, p95-HER2, is a truncated form of HER2 that lacks the extracellular domain thus rendering it incapable of binding trastuzumab [30]. However, this form retains a functional HER2 kinase domain, and its level is enhanced in nodal metastatic tissues and is inversely correlated with disease free survival [31]. Another HER2 variant is delta16 HER2, which lacks exon 16 [6]. The absence of exon 16 exposes a cysteine residue, which promotes receptor homodimerization and activation [25]. The exogenous overexpression of delta16 HER2, but not wild-type HER2, was sufficient to evoke an aggressive breast cancer phenotype in the otherwise non-tumorigenic MCF-10A mammary epithelial cell line [32]. Clinically, delta16 HER2 expression was found in approximately 90% of HER2-positive breast cancer (data from 46 human HER2-positive BCs) and is highly correlated with dissemination to local lymph nodes [25]. Delta16 HER2 has also been shown to activate entirely different signaling pathways than wild-type HER2 [32, 33], which may alter how cells respond to targeted therapies. A mutation in the kinase domain of HER2 also has been reported in cells [11] and in patient tissue samples [34], which may hinder the binding of lapatinib making it ineffective at blocking HER2 phosphorylation [11].

Other proteins have also been implicated in drug resistance of HER2-positive cancer. For instance, Wang et al. [12] reported that upregulation of estrogen receptors was primarily responsible for the initial resistance to both trastuzumab and lapatinib. However, in the later stage of resistance, cancer cells once again became reliant on HER2 signaling. Masking the HER2 receptor by a membrane-associated glycoprotein, MUC4, has been shown to prevent the binding of trastuzumab in HER2-positive JIMT1 cells [35]. In addition, MUC4 [36] and another family member MUC1-C [37], have also been shown to maintain HER2 phosphorylation in MCF7/HER2-18 (MCF7, engineered to overexpress HER2) and in BT474R and SKBR3R (resistant to trastuzumab after long term selection) cells, respectively. Lastly, increased expression of HER2 receptor ligands that can stimulate HER2 activation, has been reported in a BT474 cell line with acquired resistance to lapatinib [12].

These mechanistic studies suggest that resistant cancers still depend on HER2, while highlighting the shortcomings of monoclonal antibodies and small molecule inhibitors targeting HER2 in the resistant cells. Herein, we show that RNA interference (RNAi) by siRNA, which halts protein synthesis at the mRNA level, can potentially overcome these shortcomings. Moreover, siRNA can be designed to target mutated HER2 or HER2 variants if needed.

Our success in delivering effective doses of siHER2^d75^ to treat intrinsically resistant HCC1954 tumors [19] prompted us to test it in a model of HER2-positive cancer having acquired resistance to trastuzumab (BT474-TR) and lapatinib (BT474-LR). For HER2 siRNA screening in vitro, we used a non-specific commercial transfection agent instead of our nanoparticle delivery platform to avoid confounding siHER2 effects with those of trastuzumab, which is used as the homing target agent on our nanoparticles. Our resistant BT474-TR and BT474-LR behaved similarly to those previously reported by Wang et al. [12]; their growth was not fully inhibited by exceedingly high doses of trastuzumab and lapatinib, respectively. In contrast, siHER2^d75^ was effective in inhibiting growth of both BT474-TR and BT474-LR cells. The results translated well to the trastuzumab-resistant BT474-TRgf tumor xenografts.

In our study, we showed that cancer treated long-term with siHER2^d75^ remained sensitive to the siHER2, lapatinib and trastuzumab without significant changes in the downstream phosphorylation patterns. This suggests that HER2 signaling is still the preferred signaling pathway and that the cells remained addicted to HER2-mediated survival and proliferation. Cancer is less prone to develop acquired resistance to siHER2^d75^ treatment because it circumvents many resistance mechanisms reported for small molecule inhibitors or antibodies. Specifically, siHER2^d75^ decreases HER2 mRNA, and therefore the cells are unable to synthesize more HER2 proteins to compensate for the loss of HER2 through receptor recycling and degradation. Removing HER2 by siHER2^d75^ can also negate resistance mechanisms relying on the presence or stimulation of HER2 proteins as aforementioned. In addition, siHER2^d75^ is not susceptible to resistance caused by mutations in the kinase domain or truncation of extracellular domain of HER2 that could otherwise prevent the binding of trastuzumab and lapatinib.

In all, our results suggest that siHER2^d75^ is a promising alternative to current HER2-targeted therapies. Moreover, HER2-positive tumors that have progressed on trastuzumab or lapatinib are likely to remain treatable with siRNA against HER2 while there is minimal risk of developing further resistance to this RNAi strategy. Thus, the clinical translation of HER2 siRNA to treat resistant HER2-positive cancer is highly viable.

## MATERIALS AND METHODS

### Design and synthesis of HER2 siRNA

Seventy-six siRNA duplexes targeting the coding region of human HER2 (NM_001005862) were designed, each with 3′ UU overhangs and no other modifications. Four negative control siRNAs were purchased from commercial sources: 2 non-targeting siRNAs (AllStar Negative Control siRNA from QIAGEN and ON-TARGETplus™ Control Pool from Thermo Scientific Dharmacon®), a Luciferase siRNA and a GFP siRNA. All 76 custom HER2 siRNAs were synthesized by QIAGEN. A separate non-targeting siRNA, siSCR, was loaded onto the nanoparticle for the in vivo study.

### Cell culture and transfection of siRNA

The majority of cell lines used in this study were obtained from ATCC and have not been re-authenticated prior to use. JIMT1, EFM192A, EFM192B and EFM192C were obtained from DSMZ. 21PT, 21NT, 21MT1 and 21MT2 were obtained from Dr. Kornelia Polyak (Harvard Medical School). BT474-TRgf was obtained from Drs. Robert Kerbel (University of Toronto) and Giulio Francia (now at University of Texas at El Paso). All media and supplements were purchased from Life Technologies. The growth and medium conditions of breast cancer cell lines have been previously published [38]. Cells used in the cytotoxicity were cultured according to ATCC guidelines. Transfection was carried out using DharmaFECT-1 transfection reagent (Thermo Scientific Dharmacon) diluted in OptiMEM medium (Life Technologies). Cells were seeded 24 hours prior to transfection using DharmaFECT-1 at a final dilution of 1:200. DharmaFECT-1 and HER2 siRNA were left in the wells after transfection until assay completion.

For long-term transfection, a final concentration of 1 nM siHER2^d75^ was used throughout the 15-week period. Cells were passaged such that they never exceed 75% confluency. Transfection was repeated weekly. A second set of cells were subjected to negative control siRNA transfection under the exact same conditions. At the end of the 15-week period, both populations were taken off transfection and expanded for experiments.

### Establishment of acquired resistant BT474 cell line derivatives

To establish the trastuzumab-resistant BT474-TR, parental BT474 cells were initially maintained in RPMI-1640 complete medium with 10 μg/ml trastuzumab (purchased from OHSU Pharmacy) for 1 month and then the trastuzumab concentration was gradually increased to 30 μg/ml over the next 5 months. To generate the lapatinib-resistant BT474-LR, parental BT474 cells were initially cultured in RPMI-1640 complete medium with 50 nM lapatinib (LC Laboratories, L-4804) for 1 month and then the lapatinib concentration was gradually increased to 1 μM over the next 5 months. The BT474-TR cells were then maintained in 30 μg/ml trastuzumab, while BT474-LR in 1 μM lapatinib for subsequent studies. The resistant cell derivatives were taken off trastuzumab or lapatinib 3 days prior to seeding for experiments.

### Quantification of RNA expression

The mRNA levels of HER2 and actin control were measured using QuantiGene® 2.0 Reagent System (Affymatrix Panomics). Briefly, treated and control samples were lysed 5 days after transfection. The lysates were allowed to hybridize overnight with HER2 and actin specific probes on the capture plate. Signal amplification was achieved by further hybridization with Pre-Amplifier, Amplifier and Labeled Probe. The Labeled Probe is conjugated with alkaline phosphatases, which degrade the chemiluminescent substrate added during the last step to produce luminescence. Signals were detected on the GloMax-Multi+ Detection System (Promega). The amount of luminescence is directly proportional to the amount of mRNA present in each sample. The HER2 mRNA levels of treated and control samples were then normalized to their respective actin mRNA levels. The normalized values from treated samples were divided by the averages values of the 4 control siRNAs to determine the percentage of HER2 knockdown.

RT-PCR was used to detect the mRNA level of wild-type and delta16 splice variants. Primers were previously published [39] but the MGB quencher was used instead of TAMRA on the probes. All primers and probes were ordered from Applied Biosystems (Part of Life Technologies). Total RNA was extracted and purified using the RNeasy Kit (Qiagen). 50 ng of total RNA was used in a one-step RT-PCR reaction ran on the ABI7500 Fast Thermocycler. Cycling conditions were 50°C for 15 min, 95°C for 2 min, 40 cycles of 95°C for 15 sec and 61°C for 1 min. Data were analyzed using the delta CT method by normalizing to GAPDH.

### Cell viability assay, dose response plot and calculation of GI values

Cell viability was determined using the CellTiter-Glo assay (Promega). Cells were seeded in 96-well plates 24 h prior to transfection or drug treatment. Plates were read 3 days after lapatinib treatment or 5 days after trastuzumab treatment or siRNA transfection. Dose response data points were calculated according to the current NCI-60 DTP Human Tumor Cell Line Screen protocol. Percent growth is calculated from this formula:

$$\text{Percent Growth}=\frac{\text{Treated Viability}-\text{Viability at Time }0}{\text{Vehicle Control Viability}-\text{Viability at Time }0}\times 100$$

Dose response curves were plotted and GI values were calculated using GraphPad Prism 6 (GraphPad Software).

### Cytotoxicity of T-siHER2^d75^-NP

The cytotoxicity profile of T-siHER2^d75^-NP was evaluated in four “normal/non-tumorigenic” cell lines, HEK293, HEPG2, MCF10A, and HUVEC were purchased from ATCC and grown in media as recommended by the ATCC. The cells were seeded (cells/well in 96-well plate) at 20,000 (HEK293), 250,000 (HEPG2), 120,000 (MCF10A), and 7,500 (HUVEC), respectively, for 24 h to achieve about 80% confluency. The cells were then treated with efficacious dose of T-siHER2^d75^-NP (60 nM as siHER2^d75^, 42 μg/mL as nanoparticle, [19]) or acetaminophen (APAP, Tylenol) as a drug benchmark at 25 mM following the NCL's protocol [29]. Cell viability was measured at 24 h post-treatment with the CellTiter-Glo assay, which measures adenosine triphosphate (ATP) as an indicator of metabolically active cells, and reported as percent of the untreated control's viability.

### Immunofluorescence imaging

Cells were seeded in 96-well black wall plates (Corning® Costar®) 24 hours prior to transfection. Three days after transfection, cells were fixed in 2% paraformaldehyde for 15 minutes at room temperature and then permeabilized with 0.3% Triton-X. After blocking for one hour in 2% BSA, cells were stained overnight at 4°C with primary antibodies. Secondary antibody incubation was carried out at room temperature for one hour the next day. Nuclei were stained with Hoechst 33342 dye (Life Technologies). Plates were imaged on an Olympus IX81 Scan^R automated fluorescence microscope. Images were analyzed, and signal intensities were quantitated using the included analysis software package.

### Western blot

Cells were seeded in 6-well plates 24 hours prior to transfection or drug treatment. Samples were harvested 48 hours after drug treatment and 72 hours after transfection. Cells were lysed in RIPA buffer containing protease and phosphatase inhibitor cocktails. Running samples were prepared with 4X Novex NuPAGE LDS sample buffer (Life Technologies) and supplemented with 10% beta-mercaptoethanol. Twenty micrograms of protein were loaded per lane of 4–12% Bis-Tris NuPAGE gels (Life Technologies) and ran in MOPS buffer. Proteins were then transferred to PVDF-FL membrane (Millipore) in Tris-Glycine transfer buffer. Primary antibody incubation was carried out overnight at 4°C on a rocking platform. Secondary antibody incubation was carried out at room temperature the next day. Proteins were detected using the LI-COR Odyssey imaging system (LI-COR Biosciences). Image analysis and protein quantitation were performed with ImageJ (NCI).

### Cell cycle analysis

Cells were seeded in 10-cm dishes at 1-2 x10^6^ cells per dish. After an overnight settling period, cells were transfected with selected siRNAs. After 48 or 72 hours, cells were harvested and fixed in 70% ethanol. Cells were incubated with 50 μg/ml RNase A in PBS for 30 minutes at 37°C and then stained with 50 μg/ml propidium iodide. The cells were subsequently analyzed on a BD Calibur flow cytometer. Analysis of cell cycle results was carried out using FlowJo (Tree Star, Inc.).

### Apoptosis analysis

Apoptotic cells were detected using Yo-Pro-1 staining. Briefly, BT474 cells transfected with 10 nM siHER2 or siControl were stained with 1 μmol/L of Yo-Pro-1 dye and 10 μg/mL of Hoechst 33342 for 30 minutes at 37°C. The fluorescence dyes were detected by high content imaging (Olympus IX81 Scan^R automated fluorescence microscope). The percentage of apoptotic cells were calculated by the ratio of Yo-Pro-1 and Hoechst positive cells.

### Targeted delivery of siHER2^d75^ in a mouse model of HER2-positive breast cancer xenograft

All animals were used under an approved protocol of the Institutional Animal Care and Use Committee (IACUC) of Oregon Health and Science University (OHSU) and the experiments were carried out under the auspices of the Department of Comparative Medicine of OHSU. BT474-TRgf cells (1 × 10^7^) were injected subcutaneously into the flanks of six-week-old athymic nu/nu mice (Taconic) and allowed to grow to an average size of ∼100 mm^3^. Mice were then grouped and proceeded to receive twice a week of siHER2^d75^ or siSCR loaded on our mesoporous silica based nanoconstructs containing trastuzumab (T-NP) for targeted delivery over a period of six weeks via tail vein injection. Days of injection and doses were specified in Figure 8B. Details on the nanoconstruct design, composition, and synthesis procedures can be found in our previous report [19]. Trastuzumab was administered intravenously twice weekly at a dose of 2.5 mg/kg.

## SUPPLEMENTARY FIGURES AND TABLES


