# Supplementary material for: Therapeutic siRNA for drug-resistant HER2-positive breast cancer
Source: Oncotarget. 2016 Feb 15;7(12):14727–41. doi: 10.18632/oncotarget.7409 (PMC4924747; doi:10.18632/oncotarget.7409)
Supplement: Supplementary file 1 [file oncotarget-07-14727-s001.pdf]

SUPPLEMENTARY FIGURES AND TABLES

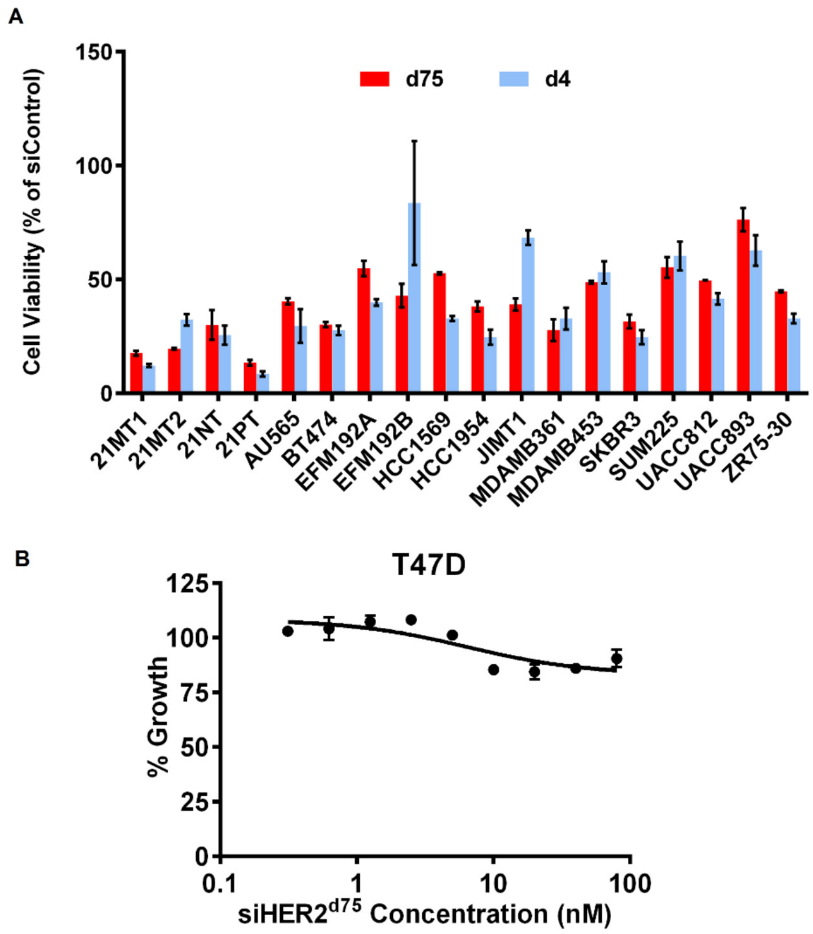

Supplementary Figure S1: A. The cell viability reduction induced by the top 2 HER2 siRNA candidates from Figure 1(A–C), measured in HER2-positive cell lines using CellTiter-Glo assay at 5 days post-transfection. All with 10 nM of siRNA delivered with DharmaFECT-1. **B.** Low off-target effect of siHER2^{d75} was confirmed in T47D (HER2-negative) cell line, in which the GI50 was not achieved even when the siHER2^{d75} concentration was increased to 80 nM.

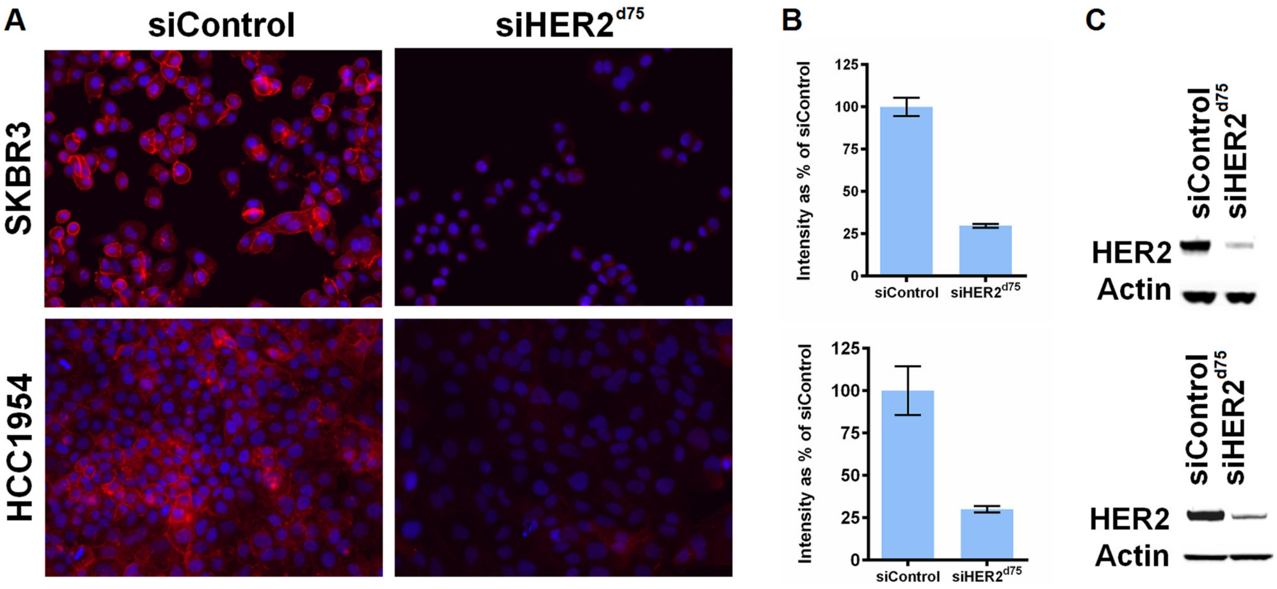

Supplementary Figure S2: Effect of siHER2^{d75} on **A.** HER2 proten reduction by immunofluorescent imaging (200x magification), **B.** corresponding quantified mean signal intensity of HER2, and **C.** HER2 protein reduction analyzed by western blot in SKBR3 and HCC1954 cells. Cells were transfected with 10 nM siHER2^{d75} or siControl for 72 h prior to analysis.

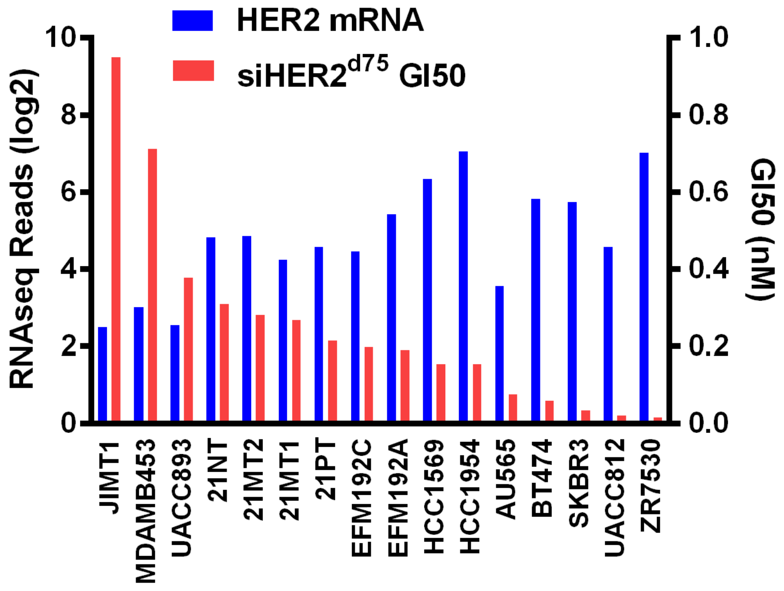

Supplementary Figure S3: Respective GI50 values of siHER2^{d75} versus HER2 mRNA expression level determined by RNAseq in the 16 HER2-positive cell lines used in the study. Experimental conditions for GI50 measurement are the same as in Figure 3.

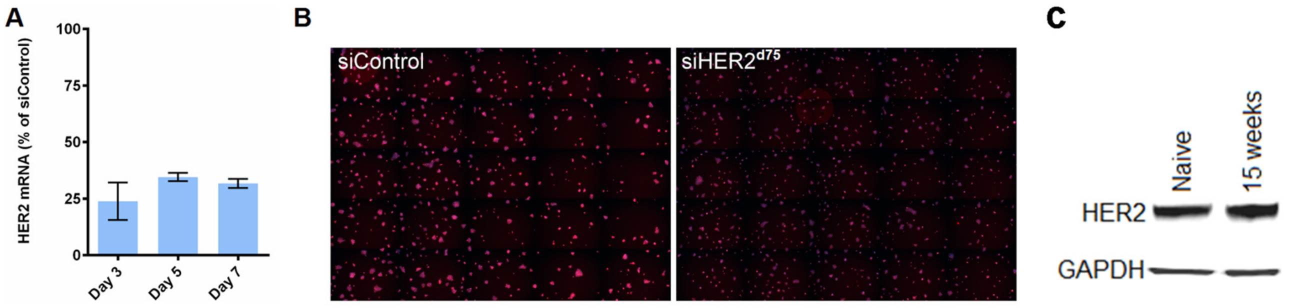

Supplementary Figure S4: A. HER2 mRNA levels in BT474 at days 3, 5 and 7 post-transfection of 1 nM siHER2^{d75}. Indicated values represent the fraction of HER2 remaining relative to siControl. **B.** HER2 levels by immunofluorescent detection in BT474 cells treated with 1 nM of siControl or siHER2^{d75} for 72 h. Image is a composite of 25 fields at 100x (each). Red = HER2, Blue = DAPI stained for nuclei. **C.** HER2 protein levels in the naïve BT474 cells (i.e., never been treated with siHER2^{d75}) and those treated continuously with 1 nM siHER2^{d75} for 15 weeks (siHER2^{d75} treatment was withdrawn for 1 week prior to western blot analysis).

Supplementary Table S1: Sequences and molecular weights of HER2 siRNAs

siRNA	Target Sequence	Molecular Weight	siRNA	Target Sequence	Molecular Weight
d1	GCCAGGUGGUGCAGGGAAA	13355	d39	GCAGAUGCGGAUCCUGAAA	13325
d2	GCUCAUCGCUCACAACCAA	13325	d40	AGAUGCGGAUCCUGAAAGA	13310
d3	GCACCCAGCUCUUUGAGGA	13340	d41	AUGCGGAUCCUGAAAGAGA	13310
d4	AUGGAGACCCGCUGAACAA	13325	d42	AAGAGACGGAGCUGAGGAA	13325
d5	GGAGACCCGCUGAACAAUA	13325	d43	GGUGAAGGUGCUUGGAUCU	13325
d6	CUUCGAAGCCUCACAGAGA	13325	d44	CGGCAGCAGAAGAUCCGGAAGUACA	17198
d7	CCUCACAGAGAUCUUGAAA	13215	d45	CCCAAAGCCAACAAAGAAA	13295
d8	UCACAGAGAUCUUGAAAGG	13333	d46	CCAAAGCCAACAAAGAAAU	13280
d9	GGGUCUUGAUCCAGCGGAA	13421	d47	GCAGAUGCGGAUCCUGAAA	13325
d10	CCCAGCUCUUUGAGGACAA	13293	d48	CGGAGCUGAGGAAGGUGAAGGUGCU	17213
d11	GCUCUUUGAGGACAACUAU	13294	d49	CGGUGCAGCUGGUGACACA	13355
d12	UCACACUGAUAGACACCAA	13295	d50	UGACACAGCUUAUGCCCUA	13310
d13	GGGAGAGAGUUCUGAGGAU	13325	d51	CUGAACUGGUGUAUGCAGA	13310
d14	GGAGAGAGUUCUGAGGAUU	13310	d52	GCAGCUGGUGACACAGCUU	13340
d15	GGAAGGACAUCUUCCACAA	13310	d53	AGACAGAGUACCAUGCAGA	13616
d16	GCUGGCUCUCACACUGAUA	13325	d54	GGUCAAGAGUCCCAACCAU	13325
d17	CGUUUGAGUCCAUGCCCAA	13325	d55	GAUGAUUGACUCUGAAUGU	13280
d18	GCAUGGAGCACUUGCGAGA	13340	d56	CGGCCAAGAUUCCGGGAGU	13355
d19	GUGCCAAUAUCCAGGAGUU	13310	d57	GGGAGUUGGUGUCUGAAUU	13310
d20	CAACCAAGAGGUGACAGCAGAGGAU	17183	d58	GCUUUGUGGUCAUCCAGAA	13303
d21	GCUCCAAGUGUUUGAGACU	13311	d59	GCUCACUGCUGGAGGACGA	13355
d22	UGUUUGAGACUCUGGAAGA	13295	d60	UGGUGGAUGCUGAGGAGUA	13325
d23	UGGAAGAGAUCACAGGUUA	13295	d61	GGUGGAUGCUGAGGAGUAU	13325
d24	GGAAGAGAUCACAGGUUAC	13310	d62	CAAAUGUUGGAUGAUUGACUCUGAA	17122
d25	GCAGUUACCAGUGCCAAUA	13304	d63	CAGCGCUUUGUGGUCAUCCAGAAUG	17179
d26	UUCCAGAACCUGCAAGUAA	13294	d64	GCCAGUCCCUUGGACAGCACCUUCU	17212
d27	CCUGGCAUUUCUGCCGGAGAGCUUU	17198	d65	CCGAUGUAUUUGAUGGUGA	13295
d28	GGGACCAGCUCUUUCGGAA	13340	d66	CCUCUGAGACUGAUGGCUA	13325
d29	GGCCAGAGGACGAGUGUGU	13355	d67	GACACUAGGGCUGGAGCCCUCUGAA	17213
d30	AGGACGAGUGUGUGGGCGA	13355	d68	GCUGGUGCCACUCUGGAAA	13340
d31	AGGAGUGCGUGGAGGAAUG	13340	d69	CGGCAGAGAACCCAGAGUA	13340
d32	GGGACCAGCUCUUUCGGAA	13340	d70	UGGAAGAGAUCACAGGUUA	13295
d33	GAGUAUGUGAAUGCCAGGCACUGUU	17168	d71	GAGACCCGCUGAACAAUAC	13326
d34	GUGUGGACCUGGAUGACAA	13325	d72	GGAGGAAUGCCGAGUACUG	13340
d35	CAGUGUGUGGCCUGUGCCCACUAUA	17198	d73	GCUCAUCGCUCACAACCAA	13324
d36	CAGCAGAAGAUCCGGAAGU	13325	d74	AACAAAGAAAUCUUAGACGAA	14536
d37	CAGAAGAUCCGGAAGUACA	13310	d75	CACGUUUGAGUCCAUGCCCAA	14611
d38	CGGAAGUACACGAUGCGGA	13340	d76	GGUGCUUGGAUCUGGCGCUUU	14626

Supplementary Table S2: Sequences of control siRNAs

SiRNA	Target Sequence	Manufacturer
Luciferase	CAAGCUGACCCUGAAGUUCUU	QIAGEN
GFP	CGUACGCGGAAUACUUCGAUU	QIAGEN
AllStar	GGGUAUCGACGAUUACAAA	QIAGEN
ON-TARGETplus	Proprietary	Thermo Scientific Dharmacon®
siSCR	UGGUUUACAUGUCGACUAA	Thermo Scientific Dharmacon®
